# Preoperative differentiation of benign and malignant gallbladder lesions using multimodal ultrasound radiomics: a retrospective study

**DOI:** 10.3389/fmed.2026.1953997

**Published:** 2026-09-16

**Authors:** Xialing Zhu, Cui Wang, Jialun Ren, Yingying Liao, Yizheng Liu, Shuping Qu, Dong Jiang

**Affiliations:** 1 Department of Ultrasound, Eastern Hepatobiliary Surgery Hospital, The Third Affiliated Hospital of Naval Medical University, Shanghai, China; 2 Department of Ultrasound, The First Affiliated Hospital of Naval Medical University, Shanghai, China; 3 Department of Comprehensive Treatment for Hepatobiliary Surgery, Eastern Hepatobiliary Surgery Hospital, The Third Affiliated Hospital of Naval Medical University, Shanghai, China

**Keywords:** contrast-enhanced ultrasound, conventional ultrasound, gallbladder lesions, machine learning, malignancy, nomogram, radiomics

## Abstract

**Objectives:**

This study aims to develop and validate a multimodal ultrasound radiomics model to differentiate benign from malignant gallbladder lesions preoperatively by integrating conventional ultrasound, contrast-enhanced ultrasound (CEUS), and clinical indicators.

**Methods:**

This retrospective study included 166 patients with histopathologically confirmed gallbladder lesions (36 malignant and 130 benign), divided in a 4:1 ratio into a cross-validation set (*n* = 133) and an independent test set (*n* = 33). A total of 37 clinical variables were collected. From each imaging modality, 513 radiomics features were extracted, normalized using parameters from the cross-validation set, and selected using sparse representation and sequential forward selection. Multilayer perceptron classifiers were developed using conventional ultrasound, CEUS, and combined features. Performance metrics and their 95% confidence intervals (CIs) were calculated, and areas under the receiver operating characteristic curves (AUCs) were compared using the paired DeLong test. Finally, a radiomics nomogram integrating the combined radiomics score and clinical indicators was constructed.

**Results:**

Among the clinical and semantic ultrasound variables, maximum lesion diameter, CEUS-derived base width, triglyceride, and high-density lipoprotein cholesterol differed significantly between benign and malignant lesions. In the cross-validation set, the conventional ultrasound, CEUS, and combined models achieved AUCs of 0.814 (95% CI: 0.689–0.938), 0.848 (95% CI: 0.764–0.932), and 0.936 (95% CI: 0.887–0.986), respectively. In the independent test set, the corresponding AUCs were 0.820 (95% CI: 0.664–0.976), 0.915 (95% CI: 0.818–1.000), and 0.920 (95% CI: 0.823–1.000). The difference between the CEUS and combined models was not significant (*P* = 0.943). At the prespecified classification threshold, the combined model achieved an accuracy of 0.909 (95% CI: 0.757–0.981), a sensitivity of 0.750 (95% CI: 0.349–0.968), a specificity of 0.960 (95% CI: 0.796–0.999), a positive predictive value of 0.857 (95% CI: 0.421–0.996), and a negative predictive value of 0.923 (95% CI: 0.749–0.991).

**Conclusions:**

The combined conventional ultrasound and CEUS radiomics model showed favorable discrimination and a balanced sensitivity–specificity profile in differentiating benign from malignant gallbladder lesions. Although its AUC was comparable to that of the CEUS radiomics model and its statistical superiority was not established, multimodal feature integration may provide complementary diagnostic information. Integration of radiomics signatures with clinical and imaging variables may support individualized preoperative risk stratification, but the incremental clinical value of this approach requires prospective multicenter validation.

## Introduction

Gallbladder lesions are increasingly detected on routine abdominal imaging, largely due to the widespread use of transabdominal ultrasonography. These lesions include a broad pathological spectrum, ranging from benign entities such as cholesterol polyps, inflammatory polyps, and adenomyomatosis to adenomas with neoplastic potential and early gallbladder carcinoma (1, 2). Although most gallbladder polypoid or space-occupying lesions are benign, accurate identification of malignant or potentially malignant lesions remains clinically important. Malignant gallbladder lesions usually require timely surgical intervention, whereas benign lesions may be managed by follow-up or selective cholecystectomy. Therefore, inaccurate preoperative risk stratification may result in either delayed treatment of malignant disease or unnecessary surgery for benign lesions (3, 4).

Conventional ultrasound is the first-line imaging modality for evaluating gallbladder lesions because it is non-invasive, inexpensive, widely available, and convenient for dynamic observation. It can provide important morphological information, including lesion size, number, location, echogenicity, surface contour, basal attachment, relationship with the gallbladder wall, associated gallstones, cholecystitis, and color Doppler flow signals (5, 6). Several conventional ultrasound features, such as larger lesion size, sessile morphology, broad base, irregular surface, gallbladder wall thickening, and intralesional vascularity, have been associated with an increased risk of neoplastic or malignant gallbladder lesions (7, 8). However, these qualitative or semi-quantitative imaging features are often insufficient for reliable discrimination, as benign and malignant lesions may exhibit overlapping sonographic appearances. Moreover, conventional ultrasound is operator-dependent and limited in its ability to characterize microvascular perfusion and subtle intralesional heterogeneity (9).

Contrast-enhanced ultrasound (CEUS) provides complementary information by dynamically displaying microvascular perfusion of gallbladder lesions. Compared with conventional ultrasound, CEUS can better depict enhancement intensity, enhancement homogeneity, vascular architecture, perfusion defects, and the relationship between the lesion base and the gallbladder wall (10, 11). These enhancement-related characteristics may reflect tumor angiogenesis, microvascular remodeling, and invasive growth. Previous studies have shown that CEUS features, including CEUS-derived base width, enhancement pattern, vascular morphology, and enhancement uniformity, may improve the differentiation of adenomatous or malignant gallbladder lesions from cholesterol polyps and other non-neoplastic lesions (12–14). Nevertheless, CEUS interpretation still relies mainly on visual assessment, and qualitative CEUS features may not fully capture the spatial complexity of lesion perfusion and internal heterogeneity.

Radiomics offers a quantitative strategy for converting medical images into high-dimensional, mineable data. By extracting first-order intensity features, texture features, and transformed-domain features, radiomics can quantify lesion heterogeneity that is difficult to recognize by visual inspection (15, 16). In recent years, radiomics has shown promising value in the diagnosis and risk stratification of hepatobiliary tumors. For gallbladder lesions, previous studies have reported that CT-based, conventional ultrasound-based, and dual-modal ultrasound radiomics models may help distinguish neoplastic from non-neoplastic polyps and improve preoperative classification (17–19). These findings suggest that radiomics may provide additional objective information beyond conventional imaging interpretation.

Machine learning further enables the integration of multidimensional imaging features and clinical indicators into individualized prediction models. By combining radiomics signatures with clinically interpretable variables, such as lesion diameter, CEUS-derived base width, surface morphology, and laboratory indicators, a predictive model or nomogram may improve diagnostic performance while maintaining clinical applicability (20, 21). Our previous study demonstrated that a CEUS-based radiomics nomogram could effectively predict neoplastic gallbladder polyps larger than 10 mm, supporting the feasibility of ultrasound radiomics for gallbladder lesion assessment (22).

Beyond radiomics, recent machine learning studies have demonstrated the feasibility of automated assessment of gallbladder lesions. Chen et al. developed a computer-aided diagnostic method using high-resolution ultrasonography, Jeong et al. reported a deep learning-based decision-support system for differentiating neoplastic from non-neoplastic gallbladder polyps, and Gupta et al. prospectively evaluated a deep learning model for ultrasound-based detection of gallbladder cancer (23–25). Together, these studies support the potential of machine learning to improve the objective classification of gallbladder lesions.

Despite these advances, three important research gaps remain. First, many previous studies focused on a single imaging modality or a limited subset of lesion subtypes. Second, the complementary value of conventional ultrasound radiomics and CEUS radiomics remains unclear. Third, relatively few studies have integrated multimodal ultrasound radiomics with clinical indicators and evaluated model performance using an independent test set.

To address these research gaps, the present study aimed to develop and validate a multimodal ultrasound radiomics model to differentiate benign from malignant gallbladder lesions. Radiomics features were extracted separately from conventional ultrasound and CEUS images, and three models were constructed based on conventional ultrasound features, CEUS features, and combined ultrasound features. The diagnostic performance of these models was evaluated in a cross-validation set and an independent test set. Furthermore, a radiomics nomogram integrating the combined radiomics score with significant clinical indicators was developed as a non-invasive, individualized tool for preoperative malignancy risk assessment.

## Materials and methods

### Study design and patients

This retrospective study included patients with gallbladder lesions who underwent preoperative conventional ultrasound and CEUS, followed by surgical resection. All lesions were histopathologically confirmed from surgical specimens, and the postoperative histopathological diagnosis was used as the reference standard. Patients were eligible for inclusion if they had (1) a gallbladder space-occupying lesion detected by conventional ultrasound; (2) available preoperative CEUS images; (3) complete clinical and laboratory data; and (4) a definitive postoperative histopathological diagnosis. Patients were excluded if they had (1) poor-quality ultrasound or CEUS images unsuitable for radiomics analysis; (2) incomplete clinical, laboratory, imaging, or histopathological information; (3) previous local treatment before the ultrasound examination; or (4) multiple lesions without a clearly identifiable target lesion.

According to the predefined inclusion and exclusion criteria, a total of 166 patients were enrolled, comprising 36 with malignant lesions and 130 with benign lesions. To evaluate model generalizability and reduce overfitting, the entire cohort was divided into a training/cross-validation set and an independent test set in a 4:1 ratio. The training/cross-validation set included 133 patients and was used for feature preprocessing, feature screening, model training, and hyperparameter optimization. The independent test set included 33 patients and was used only for final model evaluation. All steps related to feature selection and model construction were performed exclusively within the training/cross-validation set to avoid information leakage.

### Data collection

Clinical, laboratory, conventional ultrasound, and CEUS data were retrospectively collected for each patient. A total of 37 clinical and imaging-derived variables were included as candidate predictors. Clinical variables included demographic characteristics and medical history, such as sex, age, height, weight, alcohol consumption, smoking status, diabetes mellitus, hypertension, hyperlipidemia, fatty liver, and a history of malignancy.

Laboratory variables included neutrophil count, lymphocyte count, triglyceride, high-density lipoprotein cholesterol (HDL-C), low-density lipoprotein cholesterol, total cholesterol, total bilirubin, aspartate aminotransferase, alanine aminotransferase, glucose, carbohydrate antigen 19-9 (CA19-9), and carcinoembryonic antigen (CEA). Conventional ultrasound variables included lesion location, maximum lesion diameter, lesion shape, basal attachment, surface morphology, echogenicity, gallbladder wall thickening, gallstones, cholecystitis, relationship with the gallbladder wall, and color Doppler flow imaging findings. CEUS-related variables included enhancement level, enhancement pattern, and CEUS-derived base width.

The primary endpoint of this study was the benign or malignant status of the gallbladder space-occupying lesion. Malignant and benign lesions were assigned class labels 1 and 0, respectively.

### Ultrasound and contrast-enhanced ultrasound assessment

All patients underwent conventional ultrasound and CEUS examinations before treatment. Conventional ultrasound was used to evaluate the location, number, size, morphology, surface contour, internal echogenicity, basal attachment, gallbladder wall status, presence of gallstones, cholecystitis, and color Doppler blood flow signals of the target lesion. For patients with multiple gallbladder lesions, the lesion with the highest clinical suspicion or the largest measurable lesion was selected as the target lesion.

CEUS was performed after conventional ultrasound to evaluate the dynamic enhancement characteristics of the lesion. CEUS images were reviewed to assess enhancement intensity, enhancement homogeneity, vascular architecture, lesion-to-wall relationship, and CEUS-derived base width. For conventional ultrasound radiomics analysis, the image showing the maximal cross-sectional area of the target lesion, with a complete and clearly visible lesion boundary and no substantial acoustic shadowing or motion artifacts, was selected. For CEUS radiomics analysis, a representative frame was selected from the arterial-phase cine loop at or near peak lesion enhancement, generally 15–30 s after contrast agent injection. The selected CEUS frame was required to correspond as closely as possible to the conventional ultrasound imaging plane, demonstrate a complete lesion boundary, and be free of substantial respiratory or motion artifacts. When multiple adjacent frames met these criteria, the frame with the clearest lesion boundary was selected.

All images were independently reviewed by experienced ultrasound physicians who were blinded to the final diagnosis during image assessment and region-of-interest segmentation. Disagreements regarding image selection or semantic imaging features were resolved by consensus.

### Radiomics workflow

The radiomics workflow consisted of image selection, region-of-interest segmentation, image preprocessing, radiomics feature extraction, feature normalization, feature screening, feature selection, and model construction. Regions of interest (ROIs) were manually delineated along the boundary of the target lesion on representative conventional ultrasound and CEUS images. Radiomics features were extracted separately from conventional ultrasound and CEUS images. To assess the reproducibility of representative image selection, ROI delineation, and radiomics feature extraction, 30 patients, including 15 with benign lesions and 15 with malignant lesions, were selected by stratified random sampling. Two experienced ultrasound physicians independently repeated the complete image-analysis procedure, including selection of the representative conventional ultrasound image and CEUS frame, ROI delineation, and radiomics feature extraction. Both observers were blinded to the pathological diagnosis and each other's results. To assess intraobserver reproducibility, the first observer repeated the entire procedure after a 4-week interval while remaining blinded to the initial results. Interobserver intraclass correlation coefficients (ICCs) were calculated using a two-way random-effects model with absolute agreement, whereas intraobserver ICCs were calculated using a two-way mixed-effects model with absolute agreement. Radiomics features with both interobserver and intraobserver ICCs > 0.80 were considered reproducible.

For each imaging modality, a total of 513 radiomics features were extracted from the original image and eight wavelet-transformed images. Specifically, 18 histogram-based first-order features and 39 texture features were extracted from each image, resulting in 162 histogram-based features and 351 texture features. The histogram-based features described the gray-level distribution of the lesion, including energy, entropy, peak value, mean, median, range, root mean square, skewness, standard deviation, uniformity, variance, and kurtosis. The texture features were derived from the gray-level co-occurrence matrix, gray-level run-length matrix, gray-level size zone matrix, and neighborhood gray-tone difference matrix, reflecting spatial gray-level heterogeneity and textural complexity. After wavelet transformation, the same feature set was extracted from eight wavelet subimages and the original image, resulting in 513 radiomics features for each of the conventional ultrasound and CEUS images.

Before model construction, all candidate features were normalized using min-max normalization and scaled to the range of 0–1. To avoid information leakage, normalization parameters were estimated only from the training/cross-validation set and then applied to the independent test set.

### Feature screening and selection

The reproducibility of the radiomics features retained in the final models was examined using interobserver and intraobserver ICCs, with an ICC > 0.80 indicating acceptable reproducibility. For preliminary feature screening, each candidate clinical and radiomics feature was compared between benign and malignant lesions, and the corresponding *P*-value was calculated. The top 10 conventional ultrasound radiomics features and the top 10 CEUS radiomics features with the smallest *P*-values were selected for visualization using boxplots. Similarly, the top four clinical or ultrasound-derived features with the smallest *P*-values were visualized to illustrate differences in their distribution between benign and malignant lesions.

Feature selection for model construction was performed using a sparse representation-based strategy combined with sequential forward selection. First, lesion labels were represented sparsely using candidate features. The absolute values of the representation coefficients were then ranked in descending order to identify features with higher discriminative contribution. Sequential forward selection was subsequently applied to progressively construct candidate feature subsets, and the optimal feature combination was determined based on model performance in the training/cross-validation set. This strategy was used to reduce feature dimensionality and redundancy while improving model stability and generalizability.

### Model construction and evaluation

Based on the selected radiomics features, multilayer perceptron classifiers were constructed to predict malignancy. The multilayer perceptron (MLP) classifier consisted of an input layer, two hidden layers, and an output layer. The two hidden layers contained 20 and 10 neurons, respectively. Model training was performed with a batch size of 5, a learning rate of 0.0005, and a dropout rate of 0.2. The models were implemented using the PyTorch framework. Because of the imbalance between malignant and benign lesions, class weighting was applied during training to reduce classification bias.

To evaluate the contribution of different imaging modalities, three radiomics models were developed: (1) a conventional ultrasound radiomics model; (2) a CEUS radiomics model; and (3) a combined conventional ultrasound plus CEUS radiomics model. The combined radiomics score was further integrated with clinically significant predictors to construct a diagnostic radiomics nomogram for individualized prediction of malignant gallbladder lesions.

### Model evaluation

Model performance was evaluated in both the cross-validation set and the independent test set. Receiver operating characteristic (ROC) curves were generated, and the area under the curve (AUC) was calculated for each model. The 95% confidence intervals (CIs) for AUC were estimated using the DeLong method. Because predictions from the different models were obtained from the same patients, pairwise comparisons of AUCs were performed using the paired DeLong test.

Classification performance was further assessed using accuracy, sensitivity, specificity, positive predictive value (PPV), and negative predictive value (NPV). The corresponding 95% CIs were calculated using the exact Clopper–Pearson method. Class labels were assigned according to the decision rule established during model development, without re-optimizing the classification threshold in the independent test set.

Confusion matrices were generated to show the classification performance of each model. Predictive score distributions were plotted to visualize the separation between benign and malignant lesions. Decision curve analysis was performed to evaluate the clinical utility of the models across different threshold probabilities. Final model performance was primarily interpreted based on the independent test set.

The diagnostic performance of the radiomics nomogram was evaluated separately from that of the combined radiomics model. Its calibration was assessed using a calibration curve and the Hosmer–Lemeshow goodness-of-fit test.

### Statistical analysis

Statistical analyses were performed to compare clinical, laboratory, conventional ultrasound, CEUS, and radiomics features between benign and malignant lesions. Continuous variables were expressed as mean ± standard deviation or median with interquartile range, depending on data distribution. Between-group comparisons were performed using the independent-samples *t*-test or Mann–Whitney *U*-test, as appropriate. Categorical variables were expressed as counts and percentages and compared using the chi-square test or Fisher's exact test.

All model development procedures, including feature normalization, feature screening, feature selection, model training, and hyperparameter optimization, were conducted only in the training/cross-validation set. The independent test set was used only for the final evaluation. The 95% CIs for AUC were calculated using the DeLong method, whereas those for accuracy, sensitivity, specificity, positive predictive value, and negative predictive value were calculated using the exact Clopper–Pearson method. Pairwise comparisons between AUCs obtained from the same patients were performed using the paired DeLong test. The differences in AUC, corresponding 95% CIs, and two-sided *P-*values were reported. A two-sided *P*-value <0.05 was considered statistically significant.

## Results

### Baseline clinical and imaging characteristics

A total of 166 patients with gallbladder lesions were included in this study, comprising 130 with benign lesions and 36 with malignant lesions. The overall cohort was divided into a training/cross-validation set (*n* = 133) and an independent test set (*n* = 33) in a 4:1 ratio. The training/cross-validation set was used for feature screening, model construction, and parameter optimization, whereas the independent test set was used only for final model evaluation.

The baseline clinical, laboratory, conventional ultrasound, and CEUS characteristics according to lesion pathology are summarized in Table 1. Compared with benign lesions, malignant lesions showed significant differences in neutrophil count, lymphocyte count, triglyceride, HDL-C, CA19-9, CEA, maximum lesion diameter, surface morphology, complete attachment to the gallbladder wall, and CEUS-derived base width. In particular, malignant lesions had a larger maximum lesion diameter than benign lesions [12.2 (9.4–14.7) mm vs. 8.0 (6.4–9.5) mm, *P* < 0.001] and a larger CEUS-derived base width [8.2 (5.6–10.1) mm vs. 4.9 (3.5–5.6) mm, *P* < 0.001]. Malignant lesions also showed higher triglyceride levels and lower HDL-C levels than benign lesions. The baseline characteristics of the training/cross-validation set and independent test set are presented in Supplementary Table S1.

**Table 1:** Baseline clinical, laboratory, conventional ultrasound, and CEUS characteristics of patients stratified by lesion pathology.

Characteristic	All patients (*N* = 166)	Benign lesions (*n* = 130)	Malignant lesions (*n* = 36)	*P-*value
Clinical characteristics				
Sex				0.910
Male	114 (68.7)	89 (68.5)	25 (69.4)	
Female	52 (31.3)	41 (31.5)	11 (30.6)	
Age (years)	50.0 (50.0–53.3)	50.0 (50.0–54.0)	50.0 (47.8–52.5)	0.985
Height (cm)	170.0 (163.0–170.0)	170.0 (163.0–170.0)	170.0 (163.0–170.0)	0.563
Weight (kg)	65.0 (65.0–68.0)	65.0 (65.0–67.3)	65.0 (62.8–69.5)	0.844
Alcohol consumption				0.386
No	146 (88.0)	116 (89.2)	30 (83.3)	
Yes	20 (12.0)	14 (10.8)	6 (16.7)	
Smoking status				1.000
No	145 (87.3)	114 (87.7)	31 (86.1)	
Yes	21 (12.7)	16 (12.3)	5 (13.9)	
Diabetes mellitus				1.000
No	153 (92.2)	120 (92.3)	33 (91.7)	
Yes	13 (7.8)	10 (7.7)	3 (8.3)	
Hypertension				0.752
No	150 (90.4)	118 (90.8)	32 (88.9)	
Yes	16 (9.6)	12 (9.2)	4 (11.1)	
Hyperlipidemia				0.724
No	154 (92.8)	121 (93.1)	33 (91.7)	
Yes	12 (7.2)	9 (6.9)	3 (8.3)	
Fatty liver				0.810
No	127 (76.5)	100 (76.9)	27 (75.0)	
Yes	39 (23.5)	30 (23.1)	9 (25.0)	
History of malignancy				0.706
No	155 (93.4)	122 (93.8)	33 (91.7)	
Yes	11 (6.6)	8 (6.2)	3 (8.3)	
Laboratory variables				
Neutrophil count (×10^9^/L)	3.6 (3.5–3.8)	3.6 (3.5–3.8)	3.5 (3.4–3.5)	**<0.001**
Lymphocyte count (×10^9^/L)	1.8 (1.8–1.9)	1.8 (1.8–1.8)	1.9 (1.8–1.9)	**0.002**
Triglyceride (mmol/L)	1.6 (1.5–1.6)	1.5 (1.5–1.6)	2.0 (1.6–2.0)	**<0.001**
HDL-C (mmol/L)	1.4 (1.3–1.4)	1.4 (1.3–1.4)	1.1 (1.0–1.4)	**<0.001**
LDL-C (mmol/L)	3.0 (2.7–3.3)	3.0 (2.8–3.2)	3.0 (2.5–3.5)	0.586
Total cholesterol (mmol/L)	4.6 (4.2–4.9)	4.6 (4.3–4.8)	4.6 (4.0–5.1)	0.786
Total bilirubin (μmol/L)	13.3 (11.6–14.6)	13.3 (11.6–14.4)	13.4 (11.5–16.1)	0.237
AST (U/L)	20.8 (17.0–23.0)	20.8 (17.0–22.1)	20.7 (15.4–25.9)	0.664
ALT (U/L)	24.6 (18.0–27.3)	24.6 (17.4–26.9)	25.0 (18.9–29.6)	0.209
Glucose (mmol/L)	5.2 (4.8–5.3)	5.2 (4.8–5.2)	5.2 (4.7–5.6)	0.167
CA199 (U/mL)	17.5 (10.8–18.8)	18.0 (11.9–19.7)	12.2 (9.2–17.2)	**<0.001**
CEA (ng/mL)	1.8 (1.5–2.0)	1.8 (1.7–2.0)	1.5 (1.2–1.9)	**0.010**
Conventional ultrasound characteristics				
Target lesion location				0.307
Fundus	67 (40.4)	56 (43.1)	11 (30.6)	
Neck	34 (20.5)	24 (18.5)	10 (27.8)	
Body	65 (39.2)	50 (38.5)	15 (41.7)	
Maximum lesion diameter (mm)	8.5 (6.8–11.0)	8.0 (6.4–9.5)	12.2 (9.4–14.7)	**<0.001**
Lesion shape				0.145
Round	9 (5.4)	9 (6.9)	0 (0.0)	
Oval	143 (86.1)	112 (86.2)	31 (86.1)	
Irregular	14 (8.4)	9 (6.9)	5 (13.9)	
Broad-based attachment				0.282
No	48 (28.9)	35 (26.9)	13 (36.1)	
Yes	118 (71.1)	95 (73.1)	23 (63.9)	
Surface morphology				**0.015**
Smooth	144 (86.7)	118 (90.8)	26 (72.2)	
Irregular	15 (9.0)	8 (6.2)	7 (19.4)	
Nodular	7 (4.2)	4 (3.1)	3 (8.3)	
Echogenicity				0.542
Hypoechoic	82 (49.4)	64 (49.2)	18 (50.0)	
Isoechoic	72 (43.4)	58 (44.6)	14 (38.9)	
Hyperechoic	12 (7.2)	8 (6.2)	4 (11.1)	
Gallbladder wall thickening				1.000
No	149 (89.8)	116 (89.2)	33 (91.7)	
Yes	17 (10.2)	14 (10.8)	3 (8.3)	
Gallstones				0.405
No	145 (87.3)	115 (88.5)	30 (83.3)	
Yes	21 (12.7)	15 (11.5)	6 (16.7)	
Cholecystitis				0.466
No	154 (92.8)	119 (91.5)	35 (97.2)	
Yes	12 (7.2)	11 (8.5)	1 (2.8)	
Complete attachment to the gallbladder wall				**0.005**
No	154 (92.8)	125 (96.2)	29 (80.6)	
Yes	12 (7.2)	5 (3.8)	7 (19.4)	
CDFI blood flow				0.143
Absent	107 (64.5)	88 (67.7)	19 (52.8)	
Dot-like	40 (24.1)	30 (23.1)	10 (27.8)	
Strip-like	19 (11.4)	12 (9.2)	7 (19.4)	
Contrast-enhanced ultrasound characteristics				
CEUS enhancement level				0.111
Hypo-/non-enhancement	11 (6.6)	9 (6.9)	2 (5.6)	
Isoenhancement	64 (38.6)	55 (42.3)	9 (25.0)	
Hyperenhancement	91 (54.8)	66 (50.8)	25 (69.4)	
CEUS enhancement pattern				0.414
Homogeneous	116 (69.9)	93 (71.5)	23 (63.9)	
Heterogeneous	50 (30.1)	37 (28.5)	13 (36.1)	
CEUS-derived base width (mm)	5.2 (4.0–6.2)	4.9 (3.5–5.6)	8.2 (5.6–10.1)	**<0.001**

Data are presented as median (interquartile range) or *n* (%), as appropriate. *P*-values were calculated for comparisons between benign and malignant lesions.

CEUS, contrast-enhanced ultrasound; CDFI, color Doppler flow imaging; DM, diabetes mellitus; TG, triglyceride; HDL-C, high-density lipoprotein cholesterol; LDL-C, low-density lipoprotein cholesterol; AST, aspartate aminotransferase; ALT, alanine aminotransferase; CA19-9, carbohydrate antigen 19-9; CEA, carcinoembryonic antigen. Bold *P*-values indicate *P* < 0.05.

Reproducibility analysis showed that 472 of the 513 conventional ultrasound radiomics features (92.0%) and 458 of the 513 CEUS radiomics features (89.3%) had both interobserver and intraobserver ICCs > 0.80. All radiomics features retained in the final conventional ultrasound, CEUS, and combined models met the predefined reproducibility criterion. The interobserver ICCs of the model-retained features ranged from 0.812 to 0.963, and the intraobserver ICCs ranged from 0.835 to 0.978. Therefore, no model-retained radiomics features were excluded because of inadequate reproducibility.

### Conventional ultrasound radiomics features

Radiomics features extracted from conventional ultrasound images were compared between benign and malignant lesions. The top 10 conventional ultrasound radiomics features with the smallest *P*-values were selected for visualization and further analysis. These features consisted mainly of histogram-based first-order features and texture features derived from both the original and wavelet-transformed images.

The selected conventional ultrasound radiomics features included wavelet-based intensity features and texture features derived from the gray-level run-length matrix and the gray-level size zone matrix. Representative features included wavelet1 intensity median, wavelet1 gray-level run-length matrix long-run high gray-level emphasis, wavelet1 intensity skewness, wavelet1 intensity root mean square, wavelet1 gray-level run-length matrix high gray-level run emphasis, original-image gray-level run-length matrix long-run high gray-level emphasis, wavelet1 intensity mean absolute deviation, wavelet5 gray-level size zone matrix small-zone low gray-level emphasis, wavelet1 intensity mean, and original-image gray-level size zone matrix zone-size non-uniformity. The detailed feature information is presented in Table 2, and the distributions of these features are shown in Figure 1, with the corresponding feature names or defined abbreviations displayed directly on the x-axis.

**Table 2:** Top 10 conventional ultrasound radiomics features for differentiating malignant from benign gallbladder lesions.

Feature detail name	Feature class name	Abbreviation	*P*
wavelet1.itensity.media	wavelet1.itensity	WT1IMEDIA	9.05 × 10^−7^
wavelet1.textures.gray-level run-length matrix.long run high gray-level emphasis	wavelet1.texture	WT1TGLRLMLRHGE	7.37 × 10^−5^
wavelet1.itensity.h-skewness	wavelet1.itensity	WT1IHS	8.47 × 10^−5^
wavelet1.itensity.root mean square	wavelet1.itensity	WT1IRMS	8.77 × 10^−5^
wavelet1.textures.gray-level run-length matrix.high gray-level run emphasis	wavelet1.texture	WT1TGLRLMHGRE	0.000121933
image.textures.gray-level run-length matrix.long run high gray-level emphasis	image.texture	ITGLRLMLRHGE	0.000226721
wavelet1.itensity.mean absolute deviation	wavelet1.itensity	WT1IMAD	0.00024902
wavelet5.textures.gray-level size zone matrix.small zone low gray-level emphasis	wavelet5.texture	WT5TGLSZMSZLGE	0.000251608
wavelet1.itensity.h-mean	wavelet1.itensity	WT1IHM	0.000254353
image.textures.gray-level size zone matrix.zone-size nonuniformity	image.texture	ITGLSZMZSN	0.000258175

**Figure 1:**
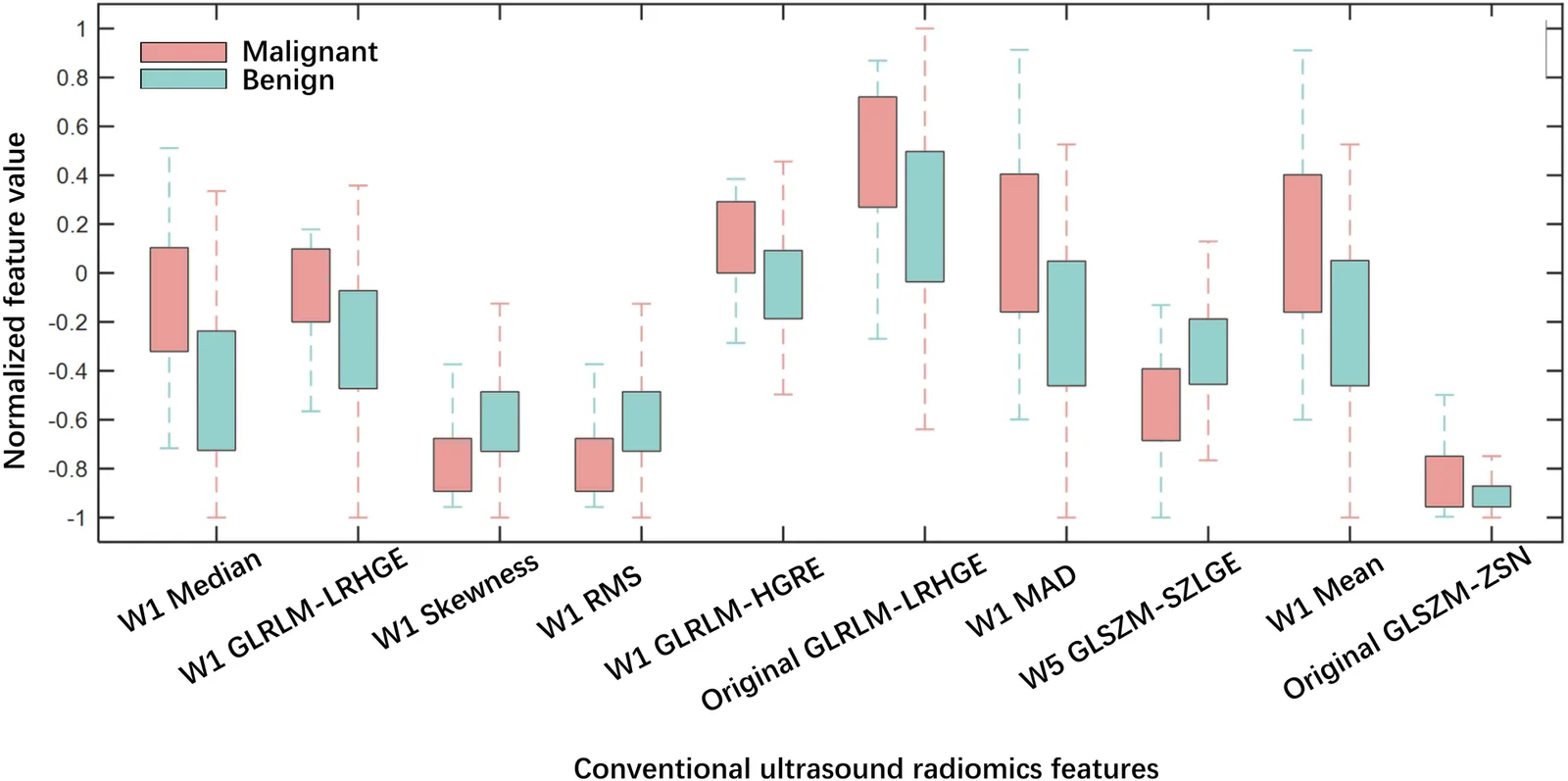
Conventional ultrasound radiomics features by lesion pathology: boxplots show the distributions of the 10 selected conventional ultrasound radiomics features. The *x*-axis directly displays the corresponding feature names or defined abbreviations, and the *y*-axis represents normalized feature values. Benign and malignant indicate histopathologically confirmed benign and malignant lesions, respectively. W1 and W5 indicate wavelet filters 1 and 5; GLRLM, gray-level run-length matrix; GLSZM, gray-level size zone matrix; LRHGE, long-run high gray-level emphasis; HGRE, high gray-level run emphasis; SZLGE, small-zone low gray-level emphasis; ZSN, zone-size non-uniformity; RMS, root mean square; MAD, mean absolute deviation.

### Contrast-enhanced ultrasound radiomics features

CEUS-derived radiomics features also differed between benign and malignant gallbladder lesions. The top 10 CEUS radiomics features with the smallest *P*-values were selected for visualization. These features were mainly wavelet-transformed intensity and texture features, suggesting that CEUS radiomics captured enhancement-related heterogeneity between benign and malignant lesions.

The selected CEUS radiomics features included wavelet4 intensity kurtosis, wavelet6 gray-level co-occurrence matrix energy, wavelet6 gray-level run-length matrix run percentage, original-image gray-level size zone matrix zone-size non-uniformity, wavelet6 gray-level co-occurrence matrix homogeneity, wavelet2 gray-level co-occurrence matrix energy, wavelet5 gray-level run-length matrix low gray-level run emphasis, wavelet5 gray-level run-length matrix short-run low gray-level emphasis, wavelet6 neighborhood gray-tone difference matrix complexity, and wavelet6 gray-level co-occurrence matrix entropy. The distribution of these CEUS radiomics features is shown in Figure 2, and detailed feature information is presented in Table 3.

**Figure 2:**
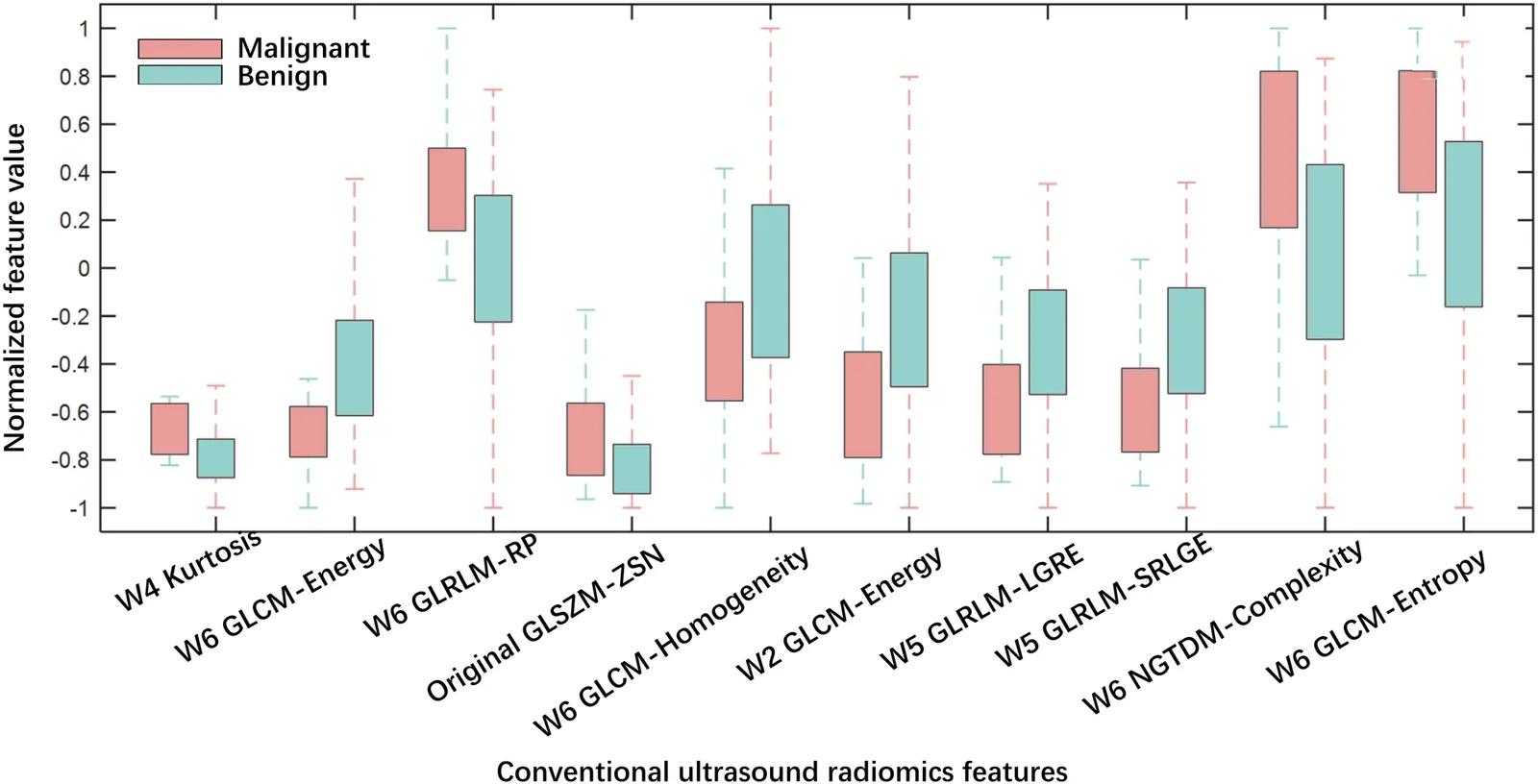
CEUS radiomics features by lesion pathology: boxplots show the distributions of the 10 selected CEUS radiomics features. The *x*-axis directly displays the corresponding feature names or defined abbreviations, and the *y*-axis represents normalized feature values. Benign and malignant indicate histopathologically confirmed benign and malignant lesions, respectively. W2, W4, W5, and W6 indicate the corresponding wavelet filters; GLCM, gray-level co-occurrence matrix; GLRLM, gray-level run-length matrix; GLSZM, gray-level size zone matrix; NGTDM, neighborhood gray-tone difference matrix; RP, run percentage; LGRE, low gray-level run emphasis; SRLGE, short-run low gray-level emphasis; ZSN, zone-size non-uniformity; CEUS, contrast-enhanced ultrasound.

**Table 3:** Top 10 CEUS radiomics features for differentiating malignant from benign gallbladder lesions.

Feature detail name	Feature class name	Abbreviation	*P*
wavelet4.itensity.h-kurtosis	wavelet4.itensity	WT4IHK	1.47 × 10^−6^
wavelet6.textures.gray-level co-occurrence matrix.energy	wavelet6.texture	WT6TGLCME	2.10 × 10^−6^
wavelet6.textures.gray-level run-length matrix.run percentage	wavelet6.texture	WT6TGLRLMRP	2.22 × 10^−5^
image.textures.gray-level size zone matrix.zone-size nonuniformity	image.texture	ITGLSZMZSN	2.48 × 10^−5^
wavelet6.textures.gray-level co-occurrence matrix.homogeneity	wavelet6.texture	WT6TGLCMH	2.55 × 10^−5^
wavelet2.textures.gray-level co-occurrence matrix.energy	wavelet2.texture	WT2TGLCME	2.59 × 10^−5^
wavelet5.textures.gray-level run-length matrix.low gray-level run emphasis	wavelet5.texture	WT5TGLRLMLGRE	5.85 × 10^−5^
wavelet5.textures.gray-level run-length matrix.short run low gray-level emphasis	wavelet5.texture	WT5TGLRLMSRLGE	6.29 × 10^−5^
wavelet6.textures.neighbourhood gray-tone difference matrix.complexity	wavelet6.texture	WT6TNGTDMCOMP	7.40 × 10^−5^
wavelet6.textures.gray-level co-occurrence matrix.entropy	wavelet6.texture	WT6TGLCME	7.46 × 10^−5^

### Clinical and ultrasound-derived features associated with malignancy

Among the 37 candidate clinical and ultrasound-derived variables, four features showed the most significant differences between benign and malignant lesions: maximum lesion diameter, CEUS-derived base width, triglyceride, and HDL-C. The corresponding *P*-values were 9.93 × 10^−12^ for maximum lesion diameter, 5.89 × 10^−9^ for CEUS-derived base width, 1.28 × 10^−7^ for triglyceride, and 8.60 × 10^−7^ for HDL-C. The distribution of these four features is shown in Figure 3, and the detailed results are listed in Table 4.

**Figure 3:**
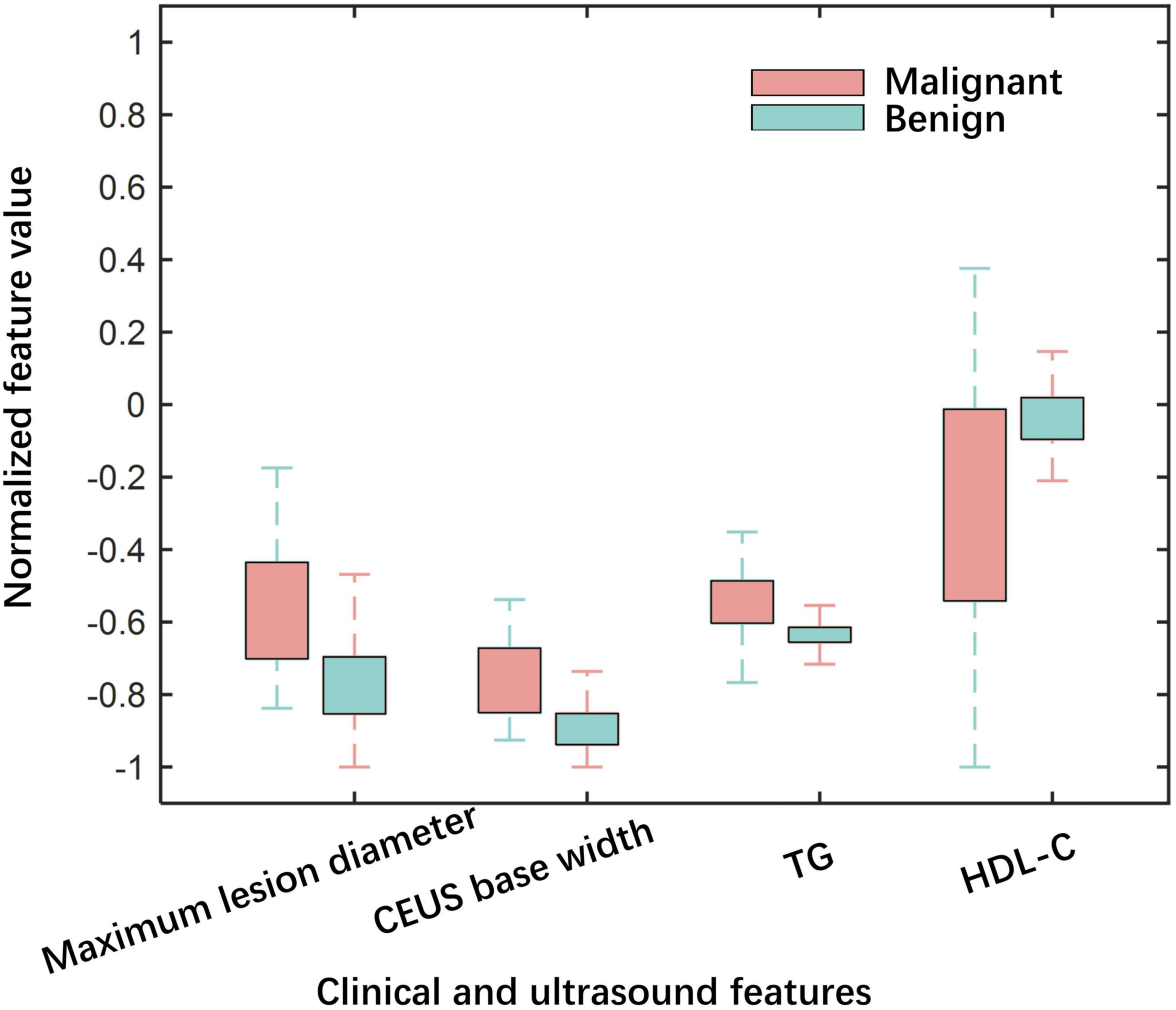
Clinical and ultrasound features by lesion pathology: boxplots show the distributions of maximum lesion diameter, CEUS-derived base width, triglyceride, and high-density lipoprotein cholesterol. The actual feature names are displayed directly on the *x*-axis, and the *y*-axis represents normalized values. Benign and malignant indicate histopathologically confirmed benign and malignant lesions, respectively. CEUS, contrast-enhanced ultrasound; TG, triglyceride; HDL-C, high-density lipoprotein cholesterol.

**Table 4:** Most discriminative clinical and ultrasound-derived features for differentiating malignant from benign gallbladder lesions.

Feature detail name	*P-*value
Maximum lesion diameter	9.93 × 10^−12^
Base width (CEUS)	5.89 × 10^−9^
TG	1.28 × 10^−7^
HDL	8.60 × 10^−7^

### Diagnostic performance of radiomics models

Three radiomics models were constructed: a conventional ultrasound radiomics model, a CEUS radiomics model, and a combined conventional ultrasound plus CEUS radiomics model. The diagnostic performance, including 95% CIs for all metrics, is summarized in Table 5.

**Table 5:** Diagnostic performance of the radiomics models and radiomics nomogram.

Dataset	Model	AUC	ACC	SEN	SPE	PPV	NPV
Cross-validation set (VA)	Conventional ultrasound	0.814 (0.689–0.938)	0.925 (0.866–0.963)	0.679 (0.476–0.841)	0.990 (0.948–1.000)	0.950 (0.751–0.999)	0.920 (0.854–0.963)
Cross-validation set (VA)	Contrast-enhanced ultrasound	0.848 (0.764–0.932)	0.902 (0.839–0.947)	0.643 (0.441–0.814)	0.971 (0.919–0.994)	0.857 (0.637–0.970)	0.911 (0.842–0.956)
Cross-validation set (VA)	Combined	0.936 (0.887–0.986)	0.932 (0.875–0.969)	0.821 (0.631–0.939)	0.962 (0.905–0.990)	0.852 (0.663–0.958)	0.953 (0.893–0.985)
Independent test set (TE)	Conventional ultrasound	0.820 (0.664–0.976)	0.818 (0.645–0.930)	0.625 (0.245–0.915)	0.880 (0.688–0.975)	0.625 (0.245–0.915)	0.880 (0.688–0.975)
Independent test set (TE)	Contrast-enhanced ultrasound	0.915 (0.818–1.000)	0.818 (0.645–0.930)	0.375 (0.085–0.755)	0.960 (0.796–0.999)	0.750 (0.194–0.994)	0.828 (0.642–0.942)
Independent test set (TE)	Combined	0.920 (0.823–1.000)	0.909 (0.757–0.981)	0.750 (0.349–0.968)	0.960 (0.796–0.999)	0.857 (0.421–0.996)	0.923 (0.749–0.991)
Independent test set (TE)	Radiomics nomogram	0.960 (0.878–1.000)	0.970 (0.842–0.999)	0.875 (0.473–0.997)	1.000 (0.863–1.000)	1.000 (0.590–1.000)	0.962 (0.804–0.999)

Data are presented as point estimates with 95% confidence intervals in parentheses. The 95% CIs for AUC were estimated using the DeLong method. The 95% CIs for accuracy, sensitivity, specificity, positive predictive value, and negative predictive value were calculated using the exact Clopper–Pearson method.

AUC, area under the receiver operating characteristic curve; ACC, accuracy; SEN, sensitivity; SPE, specificity; PPV, positive predictive value; NPV, negative predictive value; CEUS, contrast-enhanced ultrasound; CI, confidence interval.

In the cross-validation set, the conventional ultrasound, CEUS, and combined models achieved AUCs of 0.814 (95% CI: 0.689–0.938), 0.848 (95% CI: 0.764–0.932), and 0.936 (95% CI: 0.887–0.986), respectively. The combined model achieved an accuracy of 0.932 (95% CI: 0.875–0.969), a sensitivity of 0.821 (95% CI: 0.631–0.939), a specificity of 0.962 (95% CI: 0.905–0.990), a positive predictive value of 0.852 (95% CI: 0.663–0.958), and a negative predictive value of 0.953 (95% CI: 0.893–0.985).

In the independent test set, the conventional ultrasound, CEUS, and combined models achieved AUCs of 0.820 (95% CI: 0.664–0.976), 0.915 (95% CI: 0.818–1.000), and 0.920 (95% CI: 0.823–1.000), respectively. The AUC difference between the CEUS and combined models was 0.005 (95% CI: −0.132 to 0.142), and the paired DeLong test showed that this difference was not statistically significant (*P* = 0.943). At the prespecified classification threshold, the combined model achieved an accuracy of 0.909 (95% CI: 0.757–0.981), a sensitivity of 0.750 (95% CI: 0.349–0.968), a specificity of 0.960 (95% CI: 0.796–0.999), a positive predictive value of 0.857 (95% CI: 0.421–0.996), and a negative predictive value of 0.923 (95% CI: 0.749–0.991).

The CEUS radiomics model achieved a sensitivity of 0.375 (95% CI: 0.085–0.755) and specificity of 0.960 (95% CI: 0.796–0.999), whereas the combined model achieved a sensitivity of 0.750 (95% CI: 0.349–0.968) while maintaining the same specificity. Thus, although the CEUS and combined models showed comparable AUCs, the combined model demonstrated a more balanced threshold-dependent diagnostic profile. The wide CIs, particularly for sensitivity and positive predictive value, reflect the limited number of malignant lesions in the independent test set and warrant cautious interpretation.

### ROC curve analysis

ROC curve analysis was performed to compare the discriminative performance of the three radiomics models. In the cross-validation set, the AUCs of the conventional ultrasound, CEUS, and combined models were 0.814 (95% CI: 0.689–0.938), 0.848 (95% CI: 0.764–0.932), and 0.936 (95% CI: 0.887–0.986), respectively. In the independent test set, the corresponding AUCs were 0.820 (95% CI: 0.664–0.976), 0.915 (95% CI: 0.818–1.000), and 0.920 (95% CI: 0.823–1.000), respectively.

In the independent test set, the difference between the CEUS and combined models was 0.005 (95% CI: −0.132 to 0.142) and was not statistically significant according to the paired DeLong test (*P* = *0.943*). Therefore, the combined model did not demonstrate statistically superior discrimination over the CEUS radiomics model in the independent test set. The ROC curves are shown in Figure 4.

**Figure 4:**
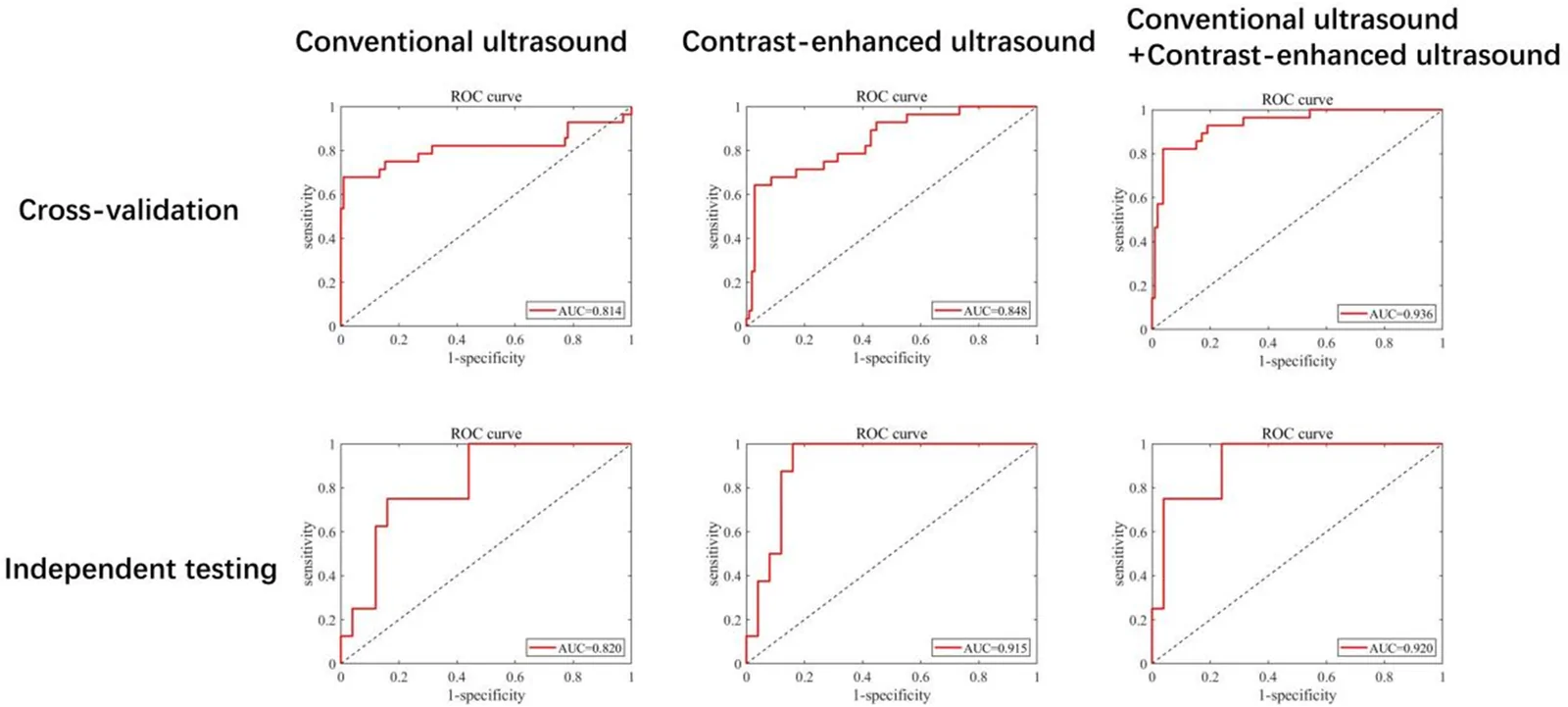
ROC curves of the three radiomics models: ROC curves compare the conventional ultrasound, CEUS, and combined ultrasound radiomics models in the cross-validation and independent test sets. The default plot titles have been removed. ROC, receiver operating characteristic; AUC, area under the curve; CEUS, contrast-enhanced ultrasound.

### Confusion matrix and predictive score analysis

Confusion matrices were generated to evaluate the classification performance of the three models. As shown in Figure 5, the combined model demonstrated a more balanced threshold-dependent classification profile in the independent test set. Compared with the CEUS radiomics model, it showed a higher point estimate of sensitivity while maintaining the same specificity; however, the wide and overlapping CIs indicate that this finding should be interpreted cautiously.

**Figure 5:**
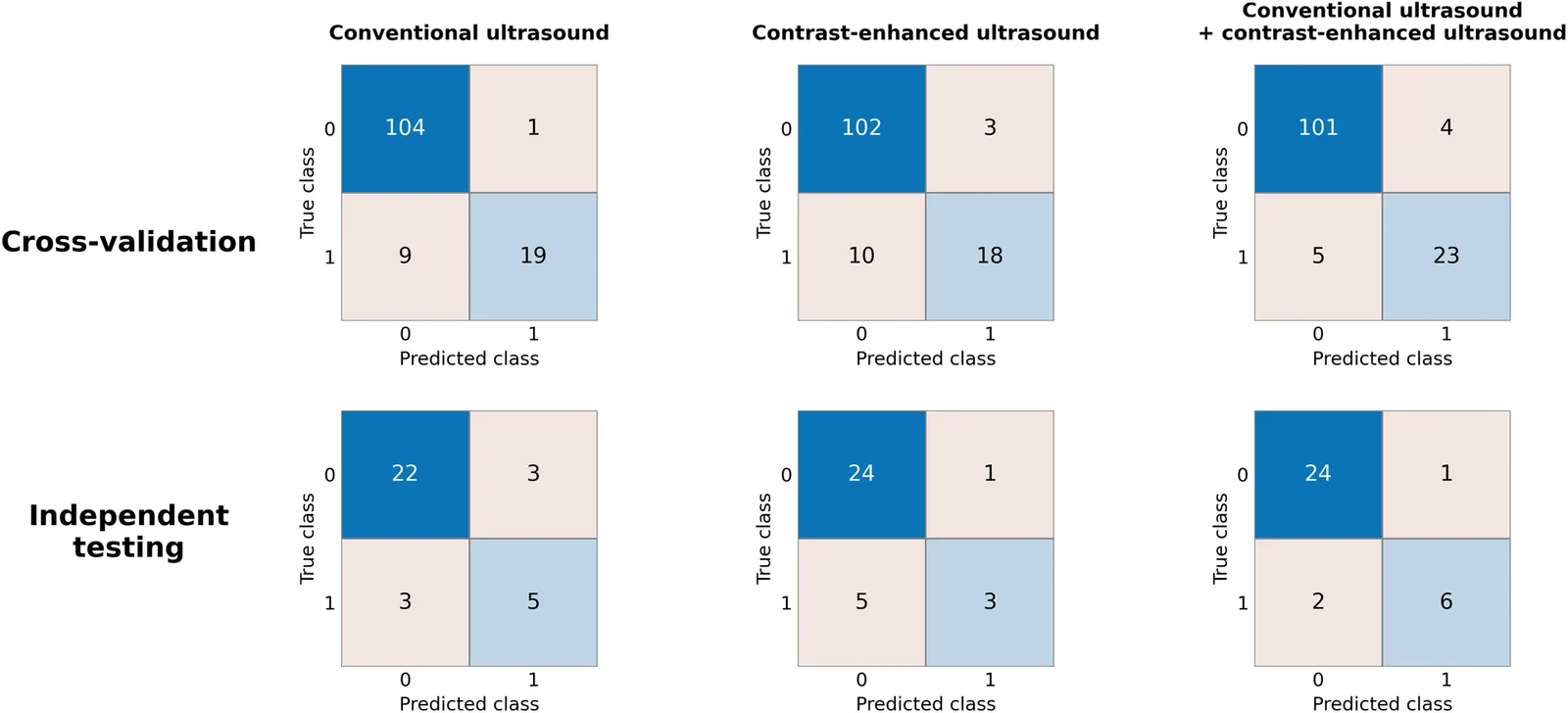
Confusion matrices of different radiomics models for differentiating benign and malignant gallbladder lesions: Confusion matrices show the classification performance of the conventional ultrasound radiomics model, CEUS radiomics model, and combined conventional ultrasound plus CEUS radiomics model in the training/cross-validation set and independent test set. The horizontal axis represents the predicted class, and the vertical axis represents the true class. Class 0 indicates benign lesions, and class 1 indicates malignant lesions. CEUS, contrast-enhanced ultrasound.

The predictive score distributions of the three models are shown in Figure 6. Compared with conventional ultrasound and CEUS radiomics models alone, the combined model showed a clearer distinction between benign and malignant lesions, with malignant lesions more frequently distributed in the higher-risk score range and benign lesions more frequently distributed in the lower-risk score range.

**Figure 6:**
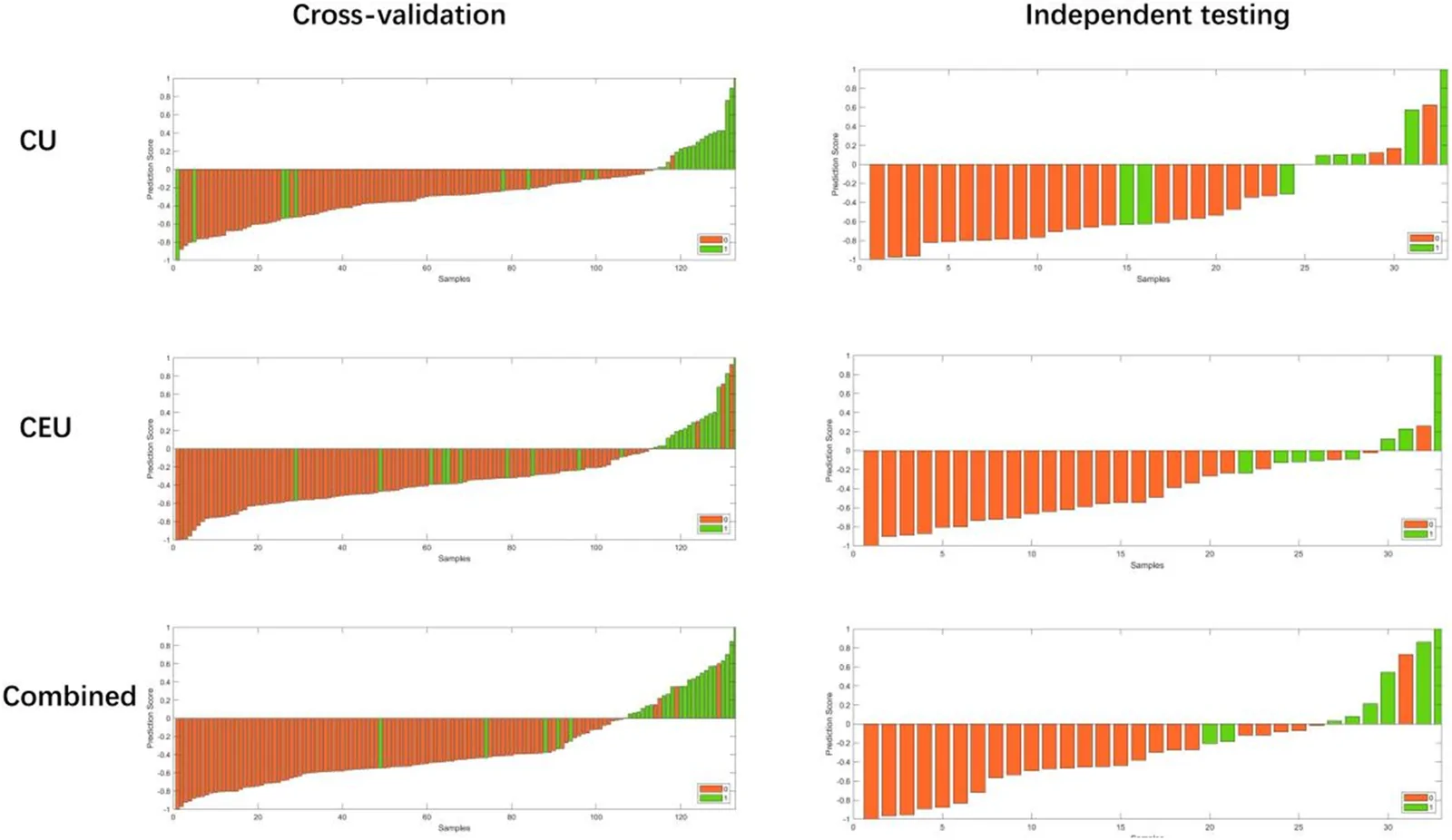
Predictive score distributions generated by different radiomics models: predictive score distributions of the conventional ultrasound radiomics model, CEUS radiomics model, and combined model are shown in the training/cross-validation set and independent test set. Each bar represents one patient, and the vertical axis represents the predicted malignancy score. The combined model showed visual separation between benign and malignant lesions. Nevertheless, because the AUC of the combined model was comparable to that of the CEUS radiomics model in the independent test set, the apparent difference in score distributions should not be interpreted as evidence of statistical superiority. CEUS, contrast-enhanced ultrasound.

### Decision curve analysis

Decision curve analysis was performed to evaluate the clinical utility of the three models. As shown in Figure 7, the combined model provided a favorable net benefit across a range of threshold probabilities in both the training/cross-validation set and the independent test set. These findings indicate that the combined model may provide a net clinical benefit across the evaluated threshold range; however, this result should be interpreted with caution given the limited size of the independent test set.

**Figure 7:**
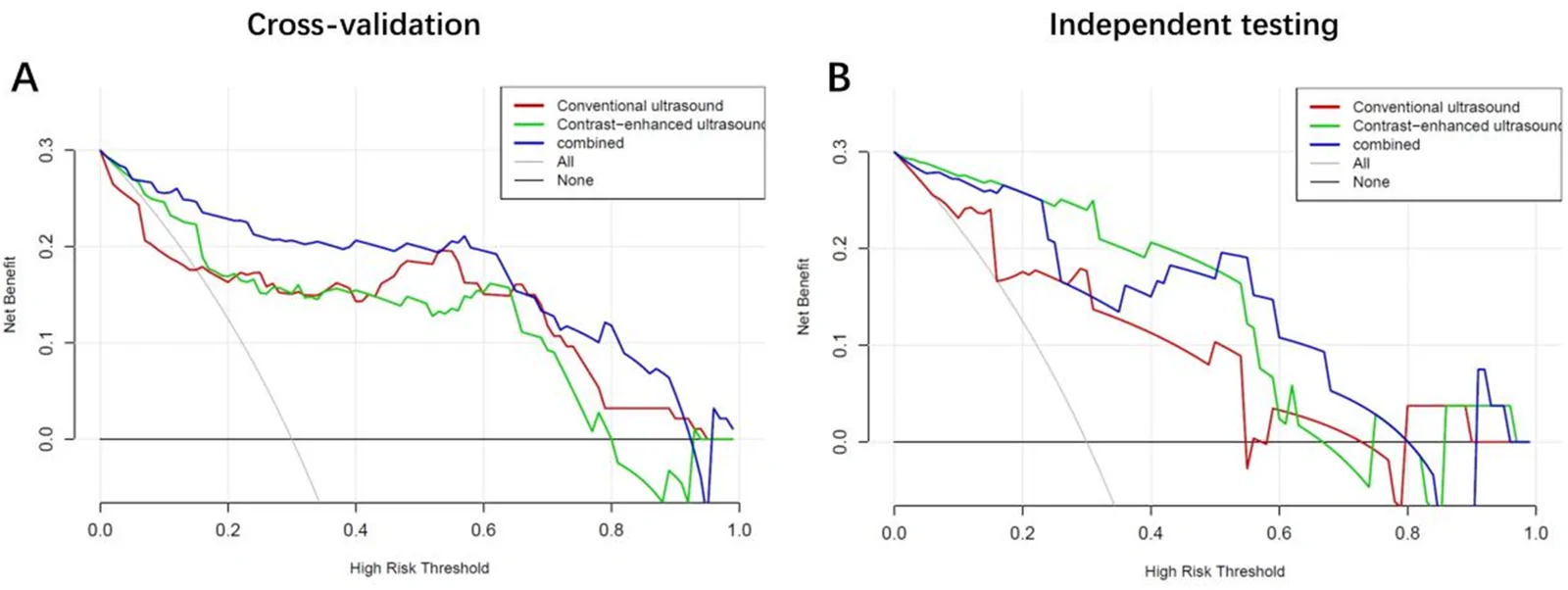
Decision curve analysis of different radiomics models: decision curve analysis was performed to evaluate the clinical utility of the conventional ultrasound radiomics model, CEUS radiomics model, and combined conventional ultrasound plus CEUS radiomics model in the training/cross-validation set and independent test set. The *x*-axis represents the threshold probability, and the *y*-axis represents the net benefit. The “All” and “None” curves represent the strategies of treating all patients and treating no patients, respectively. CEUS, contrast-enhanced ultrasound.

### Radiomics nomogram construction

A radiomics nomogram was developed by integrating the combined radiomics score with clinically relevant predictors, including maximum lesion diameter, CEUS-derived base width, triglyceride, HDL-C, complete attachment to the gallbladder wall, surface morphology, and lesion shape. As shown in Figure 8, each variable was assigned a corresponding point score, and the total score was used to estimate the individualized probability of malignancy. This nomogram provides a visual tool for preoperative risk assessment of malignant gallbladder lesions.

**Figure 8:**
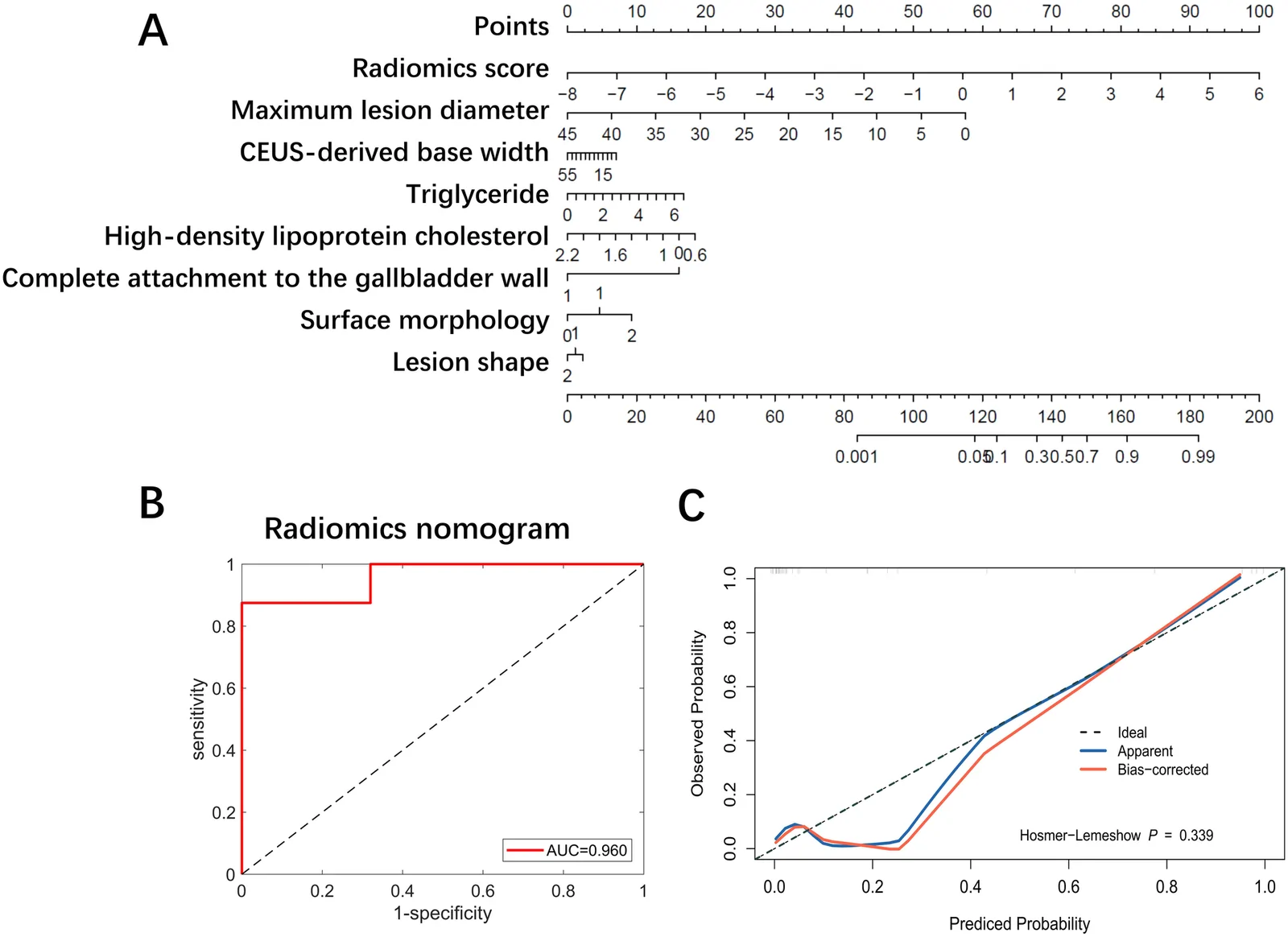
Radiomics nomogram for individualized prediction of malignant gallbladder lesions: **(A)** radiomics nomogram integrates the combined radiomics score with selected clinical and ultrasound-derived predictors, including maximum lesion diameter, CEUS-derived base width, triglyceride, high-density lipoprotein cholesterol, complete attachment to the gallbladder wall, surface morphology, and lesion shape. Each predictor is assigned a point score, and the total points are used to estimate the individualized probability of malignancy. **(B)** Receiver operating characteristic curve of the radiomics nomogram in the independent test set, showing an area under the curve of 0.960. **(C)** Calibration curve showing the agreement between the predicted and observed probabilities. The diagonal line represents perfect calibration, whereas the apparent and bootstrap bias-corrected curves represent the original and internally corrected estimates, respectively. The Hosmer–Lemeshow test showed no statistically significant evidence of lack of fit (*P* = 0.339). AUC, area under the receiver operating characteristic curve; CEUS, contrast-enhanced ultrasound.

In the independent test set, the radiomics nomogram achieved an AUC of 0.960, an accuracy of 0.970, a sensitivity of 0.875, a specificity of 1.000, a positive predictive value of 1.000, and a negative predictive value of 0.962. Compared with the combined radiomics model, the nomogram showed absolute increases of 0.040 in AUC, 0.061 in accuracy, 0.125 in sensitivity, 0.040 in specificity, 0.143 in the positive predictive value, and 0.039 in the negative predictive value. The calibration curve showed generally acceptable agreement between the predicted and observed probabilities, and the Hosmer–Lemeshow test showed no statistically significant evidence of lack of fit (*P* = 0.339). Nevertheless, these results should be interpreted with cautious due to the limited size of the independent test set.

## Discussion

In this study, we developed and internally evaluated multimodal ultrasound radiomics models to differentiate benign from malignant gallbladder lesions. The combined radiomics model showed favorable discrimination but was not statistically superior to the CEUS radiomics model in the independent test set. Its more balanced sensitivity–specificity profile at the selected threshold suggests potential complementary value rather than superior overall discrimination. This distinction is important because AUC reflects discrimination across all thresholds, whereas sensitivity and specificity depend on the selected operating threshold. Therefore, the findings support cautious interpretation of multimodal feature integration.

Accurate preoperative identification of malignant gallbladder lesions remains clinically important because treatment strategies differ substantially between benign lesions, which require surveillance, and malignant or potentially malignant lesions, which require timely intervention (1–4). Existing studies and guidelines have emphasized that lesion size, sessile morphology, broad base, and suspicious wall-related features are associated with increased malignant potential (4, 7, 8). In the present study, malignant lesions had a significantly larger maximum lesion diameter and CEUS-derived base width than benign lesions. These results are consistent with previous evidence and suggest that lesion size and basal attachment remain key features in semantic imaging for malignancy assessment. In particular, CEUS-derived base width may more clearly delineate the enhancing attachment zone between the lesion and the gallbladder wall, thereby providing more direct information on lesion–wall interaction than conventional ultrasound alone.

The three radiomics models showed different diagnostic behaviors. The conventional ultrasound radiomics model achieved AUCs of 0.814 (95% CI: 0.689–0.938) and 0.820 (95% CI: 0.664–0.976) in the cross-validation and independent test sets, respectively, indicating stable but moderate discriminative ability. Conventional ultrasound radiomics may reflect lesion morphology, internal echogenicity, surface contour, and structural heterogeneity, which are important but sometimes insufficient for identifying malignant lesions. This finding is consistent with previous ultrasound-based radiomics studies showing that radiomics can extract quantitative information from gallbladder lesions beyond routine visual interpretation (18, 19).

The CEUS radiomics model showed an AUC of 0.848 (95% CI: 0.764–0.932) in the cross-validation set and an AUC of 0.915 (95% CI: 0.818–1.000) in the independent test set, indicating that CEUS-derived radiomics features had strong discriminative potential. However, in the independent test set, the CEUS radiomics model had a sensitivity of only 0.375 (95% CI: 0.085–0.755), despite a specificity of 0.960 (95% CI: 0.796–0.999). This suggests that CEUS radiomics alone may be highly specific but may miss some malignant lesions when used as a single modality at the selected operating threshold. The wide confidence interval for sensitivity also reflects the limited number of malignant lesions in the independent test set and suggests that this result should be interpreted with caution. Previous CEUS studies have shown that enhancement intensity, enhancement pattern, vascular morphology, and CEUS-derived base width help differentiate gallbladder lesions (10–14, 22). Our results further suggest that CEUS radiomics can quantify enhancement-related heterogeneity and that its conventional ultrasound features may complement its threshold-dependent diagnostic profile.

The more balanced threshold-dependent performance of the combined model supports the potential complementary value of conventional ultrasound and CEUS. Conventional ultrasound mainly reflects lesion morphology and structural heterogeneity, whereas CEUS captures perfusion heterogeneity, vascular distribution, and enhancement-related biological behavior. These two modalities therefore provide different but complementary information. In the independent test set, the combined model showed a higher point estimate of sensitivity than the conventional ultrasound and CEUS radiomics models (0.750 vs. 0.625 and 0.375, respectively), while maintaining a high specificity of 0.960. This higher sensitivity may be clinically relevant, as missed malignant lesions can delay definitive treatment. However, given the wide and overlapping confidence intervals and the absence of a significant AUC difference between the combined and CEUS radiomics models, this potential advantage should be interpreted cautiously and requires confirmation in a larger independent cohort. Similar findings have been reported in previous dual-modal ultrasound radiomics studies, in which combined radiomics signatures showed potential complementary diagnostic value compared with single-modality models (18, 22).

The selected conventional ultrasound radiomics features were mainly histogram-based first-order features and texture features, including gray-level run-length matrix and gray-level size zone matrix features. These features characterize gray-level distribution, run-length patterns, and zone-size heterogeneity within the lesion. Malignant lesions often exhibit more heterogeneous internal architecture due to irregular cellular arrangement, stromal reaction, necrosis, or invasive growth. Such subtle heterogeneity may be difficult to assess consistently by visual inspection but can be quantitatively captured by radiomics. This may explain why the conventional ultrasound radiomics model achieved stable performance across both the cross-validation and independent test sets.

The selected CEUS radiomics features were primarily wavelet-transformed intensity and texture features, including gray-level co-occurrence matrix, gray-level run-length matrix, gray-level size zone matrix, and neighborhood gray-tone difference matrix features. These features reflect spatial heterogeneity in enhancement and textural complexity after contrast administration. The fact that many discriminative CEUS features were derived from wavelet-transformed images suggests that malignant lesions may demonstrate perfusion heterogeneity at multiple spatial scales. This result supports the value of radiomics for extracting quantitative enhancement information that subjective CEUS descriptors may not fully capture.

Triglyceride and HDL-C also differed between benign and malignant lesions. Previous studies have associated lipid metabolism and metabolic syndrome-related factors with gallbladder polyp formation and malignant potential (26, 27). Nevertheless, these lipid indicators should not be interpreted as independent diagnostic markers. Their potential value lies in providing complementary systemic information when integrated with imaging-derived predictors. These findings may also support individualized risk stratification and management of patients with gallbladder polypoid lesions (28).

The radiomics nomogram developed in this study integrated the combined radiomics score with clinically interpretable predictors, including maximum lesion diameter, CEUS-derived base width, triglyceride, HDL-C, relationship with the gallbladder wall, surface morphology, and lesion shape. This combination has potential practical translational value. The radiomics score contributes high-dimensional quantitative imaging information, whereas clinical and semantic ultrasound variables provide interpretable risk factors familiar to clinicians. Compared with purely algorithmic outputs, a nomogram can visually estimate individualized malignancy probability and may be more acceptable for clinical decision-making (20, 22, 29).

The proposed model is intended as an adjunctive decision-support tool. In practice, radiomics features could be automatically extracted from standardized ultrasound and CEUS images and combined with clinical variables to estimate individualized malignancy risk. For indeterminate lesions, this information may support decisions regarding surveillance, further imaging, or surgical consultation and potentially reduce both missed malignancies and unnecessary interventions. However, external validation, automated segmentation, and software integration are required before clinical implementation.

Decision curve analysis further supported the potential clinical utility of the combined model. The combined model provided a favorable net benefit across a range of threshold probabilities in both the cross-validation and independent test sets. This suggests that the model may help identify patients who are more likely to benefit from active intervention while reducing unnecessary treatment for benign lesions. Decision curve analysis is particularly useful for evaluating whether a prediction model provides clinical benefit beyond discrimination alone (30). Therefore, the combined model may serve as an adjunctive tool for lesions with borderline size, atypical morphology, or equivocal enhancement features. Nevertheless, its clinical utility should be confirmed in larger prospective and multicenter cohorts before routine implementation.

## Strengths

This study has several strengths. First, conventional ultrasound and CEUS radiomics features were analyzed separately and in combination, enabling direct comparison of the diagnostic contribution of each modality. Second, an independent test set was used for final evaluation, reducing the risk of overly optimistic performance estimates. Third, class weighting was applied during model training to address the imbalance between benign and malignant lesions. Fourth, model performance was evaluated comprehensively using AUC, accuracy, sensitivity, specificity, PPV, NPV, confusion matrices, predictive score distributions, and decision curve analysis. The revised analysis additionally reports 95% CIs for the diagnostic performance metrics and formally compares the AUCs of the different models, thereby avoiding conclusions based solely on numerical differences in point estimates. These procedures are consistent with current recommendations for developing and reporting prediction models (31).

## Limitations

Several limitations should be acknowledged. First, this retrospective single-center study had a limited sample size, particularly in the number of malignant lesions and patients in the independent test set. Although cross-validation, class weighting, and held-out testing were used, the findings remain preliminary. Because all patients underwent surgical resection, the cohort may have been enriched for lesions with surgical indications or suspicious imaging features, which may limit generalizability to unselected patients managed by surveillance. Larger prospective multicenter cohorts are needed for external validation. Second, ultrasound and CEUS are operator-dependent, and differences in image acquisition may affect the stability of radiomics features. Although interobserver and intraobserver reproducibility were assessed in a stratified random subset and all model-retained radiomics features showed ICCs > 0.80, the analysis was based on retrospectively stored two-dimensional images and a single representative CEUS frame. Future prospective studies should further evaluate feature stability across multiple CEUS frames, imaging planes, operators, and ultrasound systems. Third, although class weighting was used, the imbalance between benign and malignant lesions may still affect the calibration and sensitivity of the model. Although the nomogram showed generally acceptable calibration, the calibration estimates may remain unstable because of the limited sample size and should be further evaluated in larger prospective multicenter cohorts. Finally, prospective validation is needed before this nomogram can be incorporated into the routine clinical workflow.

## Conclusions

This study developed and internally evaluated conventional ultrasound, CEUS, and combined radiomics models for the preoperative differentiation of benign and malignant gallbladder lesions. In the independent test set, the combined model achieved an AUC of 0.920, an accuracy of 0.909, a sensitivity of 0.750, and a specificity of 0.960. Its AUC was comparable to that of the CEUS radiomics model (0.915; *P* = 0.943), although it showed a more balanced sensitivity–specificity profile at the selected threshold. These findings suggest that multimodal radiomics may complement conventional imaging assessment and provide a non-invasive framework for individualized preoperative malignancy risk stratification; however, prospective multicenter external validation is required before clinical implementation.

## Data Availability

The raw data supporting the conclusions of this article will be made available by the authors, without undue reservation.

## References

[1] Myers RP Shaffer EA Beck PL. Gallbladder polyps: epidemiology, natural history and management. Can J Gastroenterol. (2002) 16:187–94. 10.1155/2002/787598 11930198

[2] Kim KH. Gallbladder polyps: evolving approach to the diagnosis and management. Yeungnam Univ J Med. (2021) 38:1–9. 10.12701/yujm.2020.00213 33045805 PMC7787897

[3] Van Dooren M De Reuver PR. Gallbladder polyps and the challenge of distinguishing benign lesions from cancer. United European Gastroenterol J. (2022) 10:625–6. 10.1002/ueg2.12287 35951350 PMC9486489

[4] Foley KG Lahaye MJ Thoeni RF Soltes M Dewhurst C Barbu ST. Management and follow-up of gallbladder polyps: updated joint guidelines between the ESGAR, EAES, EFISDS and ESGE. Eur Radiol. (2022) 32:3358–68. 10.1007/s00330-021-08384-w 34918177 PMC9038818

[5] Wennmacker SZ Lamberts MP Di Martino M Drenth JPH Gurusamy KS Van Laarhoven CJHM. Transabdominal ultrasound and endoscopic ultrasound for diagnosis of gallbladder polyps. Cochrane Database Syst Rev. (2018) 8:CD012233. 10.1002/14651858.CD012233.pub2 30109701 PMC6513652

[6] Cocco G Basilico R Delli Pizzi A Cocco N Boccatonda A D’Ardes D. Gallbladder polyps ultrasound: what the sonographer needs to know. J Ultrasound. (2021) 24:131–42. 10.1007/s40477-021-00563-1 33548050 PMC8137797

[7] Fujiwara K Abe A Masatsugu T Hirano T Sada M. Effect of gallbladder polyp size on the prediction and detection of gallbladder cancer. Surg Endosc. (2021) 35:5179–85. 10.1007/s00464-020-08010-8 32974780

[8] Kamaya A Fung C Szpakowski J-L Fetzer DT Walsh AJ Alimi Y. Management of incidentally detected gallbladder polyps: society of radiologists in ultrasound consensus conference recommendations. Radiology. (2022) 305:277–89. 10.1148/radiol.213079 35787200

[9] Costa AG Guerrero VL Monforte MNG González NB Monzonís AR Maroto BC. Is ultrasonography accurate for the diagnosis of gallbladder polyps? A review of cholecystectomy specimens from patients diagnosed with gallbladder polyps over a 14-years period. Cir Esp (Engl Ed). (2023) 101:701–7. 10.1016/j.cireng.2023.02.009 37748643

[10] Zhang HP Bai M Gu JY He YQ Qiao XH Du LF. Value of contrast-enhanced ultrasound in the differential diagnosis of gallbladder lesion. World J Gastroenterol. (2018) 24:744–51. 10.3748/wjg.v24.i6.744 29456413 PMC5807677

[11] Miwa H Numata K Sugimori K Sanga K Hirotani A Tezuka S. Differential diagnosis of gallbladder polypoid lesions using contrast-enhanced ultrasound. Abdom Radiol (NY). (2019) 44:1367–78. 10.1007/s00261-018-1833-4 30478647

[12] Zhu L Han P Lee R Jiang B Jiao Z Li N. Contrast-enhanced ultrasound to assess gallbladder polyps. Clin Imaging. (2021) 78:8–13. 10.1016/j.clinimag.2021.02.015 33706069

[13] Wang X Zhu J Liu Y Liu Y Che D Niu S. Conventional ultrasound combined with contrast-enhanced ultrasound in differential diagnosis of gallbladder cholesterol and adenomatous polyps (1–2 cm). J Ultrasound Med. (2022) 41:617–26. 10.1002/jum.15740 33938029

[14] Fei X Cheng Z Zhu L Han P Li N Jiao Z. A practical contrast-enhanced ultrasound risk prediction of gallbladder polyp: differentiation of adenoma from cholesterol polyp lesion. Abdom Radiol (NY). (2025) 50:747–56. 10.1007/s00261-024-04566-4 39254706

[15] Mayerhoefer ME Materka A Langs G Häggström I Szczypiński P Gibbs P. Introduction to radiomics. J Nucl Med. (2020) 61:488–95. 10.2967/jnumed.118.222893 32060219 PMC9374044

[16] Yao Z Dong Y Wu G Zhang Q Yang D Yu J-H. Preoperative diagnosis and prediction of hepatocellular carcinoma: radiomics analysis based on multi-modal ultrasound images. BMC Cancer. (2018) 18:1089. 10.1186/s12885-018-5003-4 30419849 PMC6233500

[17] Yang X Liu Y Guo Y Chai R Niu M Xu K. Utility of radiomics based on contrast-enhanced CT and clinical data in the differentiation of benign and malignant gallbladder polypoid lesions. Abdom Radiol (NY). (2020) 45:2449–58. 10.1007/s00261-020-02461-2 32166337

[18] Yuan HX Wang C Tang CY You QQ Zhang Q Wang WP. Differential diagnosis of gallbladder neoplastic polyps and cholesterol polyps with radiomics of dual modal ultrasound: a pilot study. BMC Med Imaging. (2023) 23:26. 10.1186/s12880-023-00982-y 36747143 PMC9901123

[19] Zhang X Wang J Wu B Li T Jin L Wu Y. A nomogram-based model and ultrasonic radiomic features for gallbladder polyp classification. J Gastroenterol Hepatol. (2022) 37:1380–8. 10.1111/jgh.15841 35357026

[20] Han S Liu Y Li X Jiang X Li B Zhang C. Development and validation of a preoperative nomogram for predicting benign and malignant gallbladder polypoid lesions. Front Oncol. (2022) 12:800449. 10.3389/fonc.2022.800449 35402267 PMC8990775

[21] Li Q Dou M Zhang J Jia P Wang X Lei D. A Bayesian network model to predict neoplastic risk for patients with gallbladder polyps larger than 10 mm based on preoperative ultrasound features. Surg Endosc. (2023) 37:5453–63. 10.1007/s00464-023-10056-3 37041283

[22] Jiang D Qian Y Gu Y Wang R Yu H Wang Z. Establishing a radiomics model using contrast-enhanced ultrasound for preoperative prediction of neoplastic gallbladder polyps exceeding 10 mm. Eur J Med Res. (2025) 30:66. 10.1186/s40001-025-02292-1 39901203 PMC11789348

[23] Chen T Tu S Wang H Liu X Li F Jin W. Computer-aided diagnosis of gallbladder polyps based on high resolution ultrasonography. Comput Methods Programs Biomed. (2020) 185:105118. 10.1016/j.cmpb.2019.105118 31671340

[24] Jeong Y Kim JH Chae H-D Park S-J Bae JS Joo I. Deep learning-based decision support system for the diagnosis of neoplastic gallbladder polyps on ultrasonography: preliminary results. Sci Rep. (2020) 10:7700. 10.1038/s41598-020-64205-y 32382062 PMC7205977

[25] Gupta P Basu S Rana P Dutta U Soundararajan R Kalage D. Deep-learning enabled ultrasound based detection of gallbladder cancer in northern India: a prospective diagnostic study. Lancet Reg Health Southeast Asia. (2024) 24:100279. 10.1016/j.lansea.2023.100279 38756152 PMC11096661

[26] Yu Z Yang C Bai X Yao G Qian X Gao W. Risk factors for cholesterol polyp formation in the gallbladder are closely related to lipid metabolism. Lipids Health Dis. (2021) 20:26. 10.1186/s12944-021-01452-6 33752687 PMC7983281

[27] Deng Z Xuan Y Li X Crawford WJ Yuan Z Chen Z. Effect of metabolic syndrome components on the risk of malignancy in patients with gallbladder lesions. J Cancer. (2021) 12:1531–7. 10.7150/jca.54617 33531998 PMC7847661

[28] Tian F Ma Y- Liu Y- Liu W Hong T He X- Management strategy for gallbladder polypoid lesions: results of a 5-year single-center cohort study. Dig Surg. (2022) 39:263–73. 10.1159/000529221 36696883

[29] Zhang D Li Q Zhang X Jia P Wang X Geng X. Establishment of a nomogram prediction model for long diameter 10–15 mm gallbladder polyps with malignant tendency. Surgery. (2021) 170:664–72. 10.1016/j.surg.2021.04.035 34090677

[30] Vickers AJ Elkin EB. Decision curve analysis: a novel method for evaluating prediction models. Med Decis Making. (2006) 26:565–74. 10.1177/0272989X06295361 17099194 PMC2577036

[31] Collins GS Reitsma JB Altman DG Moons KGM. Transparent reporting of a multivariable prediction model for individual prognosis or diagnosis (TRIPOD): the TRIPOD statement. Ann Intern Med. (2015) 162:55–63. 10.7326/M14-0697 25560714

