# Supplementary material for: Preoperative differentiation of benign and malignant gallbladder lesions using multimodal ultrasound radiomics: a retrospective study
Source: Front Med (Lausanne). 2026 Sep 16;13:1953997. doi: 10.3389/fmed.2026.1953997 (PMC13623621; doi:10.3389/fmed.2026.1953997)
Supplement: Supplementary file 1 [file Table1.docx]

Supplementary Table 1. Baseline characteristics of the cross-validation and independent test sets.

| **Characteristic** | **All patients (N = 166)** | **Cross-validation set (n = 133)** | **Independent test set (n = 33)** | ***P* value** |
| --- | --- | --- | --- | --- |
| **Clinical characteristics** | | | | |
| Sex |  |  |  | 1.000 |
| Male | 114 (68.7) | 91 (68.4) | 23 (69.7) |  |
| Female | 52 (31.3) | 42 (31.6) | 10 (30.3) |  |
| Age (years) | 50.0 (50.0-53.3) | 50.0 (50.0-54.0) | 50.0 (50.0-50.5) | 0.985 |
| Height (cm) | 170.0 (163.0-170.0) | 170.0 (163.0-170.0) | 167.0 (161.0-170.0) | 0.312 |
| Weight (kg) | 65.0 (65.0-68.0) | 65.0 (65.0-68.0) | 65.0 (65.0-65.5) | 0.960 |
| Alcohol consumption |  |  |  | 0.080 |
| No | 146 (88.0) | 120 (90.2) | 26 (78.8) |  |
| Yes | 20 (12.0) | 13 (9.8) | 7 (21.2) |  |
| Smoking status |  |  |  | 1.000 |
| No | 145 (87.3) | 116 (87.2) | 29 (87.9) |  |
| Yes | 21 (12.7) | 17 (12.8) | 4 (12.1) |  |
| Diabetes mellitus |  |  |  | 1.000 |
| No | 153 (92.2) | 122 (91.7) | 31 (93.9) |  |
| Yes | 13 (7.8) | 11 (8.3) | 2 (6.1) |  |
| Hypertension |  |  |  | 0.318 |
| No | 150 (90.4) | 122 (91.7) | 28 (84.8) |  |
| Yes | 16 (9.6) | 11 (8.3) | 5 (15.2) |  |
| Hyperlipidemia |  |  |  | 1.000 |
| No | 154 (92.8) | 123 (92.5) | 31 (93.9) |  |
| Yes | 12 (7.2) | 10 (7.5) | 2 (6.1) |  |
| Fatty liver |  |  |  | 0.303 |
| No | 127 (76.5) | 104 (78.2) | 23 (69.7) |  |
| Yes | 39 (23.5) | 29 (21.8) | 10 (30.3) |  |
| History of malignancy |  |  |  | 0.123 |
| No | 155 (93.4) | 122 (91.7) | 33 (100.0) |  |
| Yes | 11 (6.6) | 11 (8.3) | 0 (0.0) |  |
| **Laboratory variables** | | | | |
| Neutrophil count (×10^9/L) | 3.6 (3.5-3.8) | 3.6 (3.5-3.8) | 3.6 (3.4-3.8) | 0.231 |
| Lymphocyte count (×10^9/L) | 1.8 (1.8-1.9) | 1.8 (1.8-1.9) | 1.8 (1.8-2.0) | 0.234 |
| Triglyceride (mmol/L) | 1.6 (1.5-1.6) | 1.6 (1.5-1.7) | 1.5 (1.4-1.6) | 0.477 |
| HDL-C (mmol/L) | 1.4 (1.3-1.4) | 1.4 (1.2-1.4) | 1.4 (1.3-1.5) | 0.100 |
| LDL-C (mmol/L) | 3.0 (2.7-3.3) | 3.0 (2.7-3.3) | 3.0 (2.9-3.5) | 0.157 |
| Total cholesterol (mmol/L) | 4.6 (4.2-4.9) | 4.6 (4.2-4.8) | 4.6 (4.6-5.2) | **0.013** |
| Total bilirubin (μmol/L) | 13.3 (11.6-14.6) | 13.3 (11.8-14.7) | 13.3 (11.3-13.3) | 0.099 |
| AST (U/L) | 20.8 (17.0-23.0) | 20.8 (16.6-24.0) | 20.8 (17.2-21.3) | 0.985 |
| ALT (U/L) | 24.6 (18.0-27.3) | 24.6 (17.6-27.7) | 24.6 (20.4-27.0) | 0.732 |
| Glucose (mmol/L) | 5.2 (4.8-5.3) | 5.2 (4.8-5.4) | 5.2 (4.9-5.2) | 0.724 |
| CA19-9 (U/mL) | 17.5 (10.8-18.8) | 17.2 (11.1-18.6) | 18.0 (9.2-19.3) | 0.712 |
| CEA (ng/mL) | 1.8 (1.5-2.0) | 1.8 (1.6-2.0) | 1.8 (1.3-1.9) | 0.280 |
| **Conventional ultrasound characteristics** | | | | |
| Target lesion location |  |  |  | 0.292 |
| Fundus | 67 (40.4) | 55 (41.4) | 12 (36.4) |  |
| Neck | 34 (20.5) | 24 (18.0) | 10 (30.3) |  |
| Body | 65 (39.2) | 54 (40.6) | 11 (33.3) |  |
| maximum lesion diameter (mm) | 8.5 (6.8-11.0) | 8.2 (6.6-10.2) | 9.7 (7.5-12.4) | 0.059 |
| Lesion shape |  |  |  | 0.431 |
| Round | 9 (5.4) | 8 (6.0) | 1 (3.0) |  |
| Oval | 143 (86.1) | 112 (84.2) | 31 (93.9) |  |
| Irregular | 14 (8.4) | 13 (9.8) | 1 (3.0) |  |
| Broad-based attachment |  |  |  | 0.056 |
| No | 48 (28.9) | 34 (25.6) | 14 (42.4) |  |
| Yes | 118 (71.1) | 99 (74.4) | 19 (57.6) |  |
| Surface morphology |  |  |  | 1.000 |
| Smooth | 144 (86.7) | 115 (86.5) | 29 (87.9) |  |
| Irregular | 15 (9.0) | 12 (9.0) | 3 (9.1) |  |
| Nodular | 7 (4.2) | 6 (4.5) | 1 (3.0) |  |
| Echogenicity |  |  |  | 0.465 |
| Hypoechoic | 82 (49.4) | 67 (50.4) | 15 (45.5) |  |
| Isoechoic | 72 (43.4) | 58 (43.6) | 14 (42.4) |  |
| Hyperechoic | 12 (7.2) | 8 (6.0) | 4 (12.1) |  |
| Gallbladder wall thickening |  |  |  | 0.336 |
| No | 149 (89.8) | 121 (91.0) | 28 (84.8) |  |
| Yes | 17 (10.2) | 12 (9.0) | 5 (15.2) |  |
| Gallstones |  |  |  | 0.572 |
| No | 145 (87.3) | 117 (88.0) | 28 (84.8) |  |
| Yes | 21 (12.7) | 16 (12.0) | 5 (15.2) |  |
| Cholecystitis |  |  |  | 0.463 |
| No | 154 (92.8) | 122 (91.7) | 32 (97.0) |  |
| Yes | 12 (7.2) | 11 (8.3) | 1 (3.0) |  |
| Complete attachment to gallbladder wall |  |  |  | 1.000 |
| No | 154 (92.8) | 123 (92.5) | 31 (93.9) |  |
| Yes | 12 (7.2) | 10 (7.5) | 2 (6.1) |  |
| CDFI blood flow |  |  |  | 0.838 |
| Absent | 107 (64.5) | 84 (63.2) | 23 (69.7) |  |
| Dot-like | 40 (24.1) | 33 (24.8) | 7 (21.2) |  |
| Strip-like | 19 (11.4) | 16 (12.0) | 3 (9.1) |  |
| **Contrast-enhanced ultrasound characteristics** | | | | |
| CEUS enhancement level |  |  |  | 0.904 |
| Hypo-/non-enhancement | 11 (6.6) | 9 (6.8) | 2 (6.1) |  |
| Isoenhancement | 64 (38.6) | 50 (37.6) | 14 (42.4) |  |
| Hyperenhancement | 91 (54.8) | 74 (55.6) | 17 (51.5) |  |
| CEUS enhancement pattern |  |  |  | 0.980 |
| Homogeneous | 116 (69.9) | 93 (69.9) | 23 (69.7) |  |
| Heterogeneous | 50 (30.1) | 40 (30.1) | 10 (30.3) |  |
| CEUS-derived base width (mm) | 5.2 (4.0-6.2) | 5.1 (3.9-6.2) | 5.6 (4.1-6.7) | 0.378 |

.Note: Data are presented as median (interquartile range) or n (%), as appropriate. P values were calculated for comparisons between the cross-validation set and the independent test set. CEUS, contrast-enhanced ultrasound; CDFI, color Doppler flow imaging; DM, diabetes mellitus; TG, triglyceride; HDL-C, high-density lipoprotein cholesterol; LDL-C, low-density lipoprotein cholesterol; AST, aspartate aminotransferase; ALT, alanine aminotransferase; CA19-9, carbohydrate antigen 19-9; CEA, carcinoembryonic antigen. Bold P values indicate *P*<0.05.
